# Latent tuberculosis infection, tuberculin skin test and vitamin D status in contacts of tuberculosis patients: a cross-sectional and case-control study

**DOI:** 10.1186/1471-2334-11-349

**Published:** 2011-12-15

**Authors:** Alberto Arnedo-Pena, José Vicente Juan-Cerdán, Angeles Romeu-Garcia, Daniel Garcia-Ferrer, Rita Holguín-Gómez, Jesús Iborra-Millet, Concepción Herrero-Carot, María Jesús Sanchis Piñana, Juan Bellido-Blasco, José Antonio Ferrero-Vega, Lourdes Safont Adsuara, Esther Silvestre Silvestre, Noemi Meseguer Ferrer, Vicenta Rodrigo Bartual

**Affiliations:** 1 Epidemiology Division. Public Health Center Castellón, Spain; 2 Laboratory of Biochemical. Hospital General. Castellón, Spain; 3 Preventive Medicine. Hospital La Plana. Vila-real, Spain; 4 CIBER of Epidemiology and Public Health (CIBERESP), Barcelona, Spain; 5 Preventive Medicine. Hospital Provincial. Castellón, Spain

**Keywords:** Tuberculosis, Vitamin D, Latent tuberculosis infection, Tuberculin skin test conversion, Case-control study

## Abstract

**Background:**

Deficient serum vitamin D levels have been associated with incidence of tuberculosis (TB), and latent tuberculosis infection (LTBI). However, to our knowledge, no studies on vitamin D status and tuberculin skin test (TST) conversion have been published to date. The aim of this study was to estimate the associations of serum 25-hydroxyvitamin D_3 _(25[OH]D) status with LTBI prevalence and TST conversion in contacts of active TB in Castellon (Spain).

**Methods:**

The study was designed in two phases: cross-sectional and case-control. From November 2009 to October 2010, contacts of 42 TB patients (36 pulmonary, and 6 extra-pulmonary) were studied in order to screen for TB. LTBI and TST conversion cases were defined following TST, clinical, analytic and radiographic examinations. Serum 25(OH)D levels were measured by electrochemiluminescence immunoassay (ECLIA) on a COBAS^® ^410 ROCHE^® ^analyzer. Logistic regression models were used in the statistical analysis.

**Results:**

The study comprised 202 people with a participation rate of 60.1%. Only 20.3% of the participants had a sufficient serum 25(OH)D (≥ 30 ng/ml) level. In the cross-sectional phase, 50 participants had LTBI and no association between LTBI status and serum 25(OH)D was found. After 2 months, 11 out of 93 negative LTBI participants, without primary prophylaxis, presented TST conversion with initial serum 25(OH)D levels: a:19.4% (7/36): < 20 ng/ml, b:12.5% (4/32):20-29 ng/ml, and c:0%(0/25) ≥ 30 ng/ml. A sufficient serum 25(OH)D level was a protector against TST conversion a: Odds Ratio (OR) = 1.00; b: OR = 0.49 (95% confidence interval (CI) 0.07-2.66); and c: OR = 0.10 (95% CI 0.00-0.76), trends p = 0.019, adjusted for high exposure and sputum acid-fast bacilli positive index cases. The mean of serum level 25(OH)D in TST conversion cases was lower than controls,17.5 ± 5.6 ng/ml versus 25.9 ± 13.7 ng/ml (p = 0.041).

**Conclusions:**

The results suggest that sufficient serum 25(OH)D levels protect against TST conversion.

## Background

The hypothesis of vitamin D (VitD) deficiency as a risk factor for tuberculosis (TB) was postulated by Peter Davies, based on epidemiological studies of immigrants from India who developed active TB in the United Kingdom [1]. Low levels of VitD in blood are associated with TB [2-4], and hypovitaminosis D is frequent in patients with TB [5] and children with latent tuberculosis infection (LTBI) [6]. In addition, epidemiological studies of geographic or seasonal variations of TB incidence [7-11] and differences in the efficacy of the *Micobacterium bovis *bacillus Calmette-Guérin (BCG) vaccine according to latitude, suggest that a reduction of VitD levels could increase TB risk [12,13]. Furthermore, in vitro studies [14] and a meta-analysis of studies of vitamin D receptor genetic polymorphisms support the VitD hypothesis [15]. However, there are controversies about the actions of VitD on the infection: Bruce and coauthors indicated [16] that: "The evidence does not support a positive or negative role for VitD in host resistance to infection", an alternative VitD action has been proposed [17], other factors apart from VitD could be important in relation to the seasonality of TB incidence [18-20], and the VitD hypothesis continues to be questioned [21].

It has been indicated that the hypothesis of VitD deficiency as a risk factor of TB could be tested by in vitro studies of immune responses or by case-control studies [22]. In studies where the cases are patients with active TB, it is difficult to differentiate whether low VitD levels are a cause or a consequence of the disease [23]. The study of LTBI in relation to VitD levels using a cross-sectional design presents a similar problem with positive LTBI cases [24,25]. The study of LTBI in contacts of TB patients and potential tuberculin skin test (TST) conversions could be considered as an alternative design. To our knowledge, no studies of VitD status and TST conversion have been published to date. The objective of our study was therefore to estimate associations of VitD status with LTBI prevalence and TST conversion in contacts of TB patients.

## Methods

The study was designed in two phases: the first phase was cross-sectional and the second, a case-control study. The period of study was from November 2009 to October 2010. The study involved two health departments in the province of Castellón (Spain), located on the east cost of the Iberia Peninsula at latitude 39° N. The two departments have population of 470,000. The mean annual hours of sunshine was 2689 for the period 1971-2000 according to data from the Ministry for the Environment http://www.aemet.es/es/elclima. VitD from sun exposure is considered to be scarce during the autumn and winter. The median annual rates of pulmonary and extra-pulmonary TB for years 2006-2010, were 13.4 per 100,000 inhabitants and 1.9 per 100,000 inhabitants, respectively, and the proportion of foreign-born cases was 44.6%. Although these incidences could be considered low, they are higher than most other European countries. In the study period, 53 cases of pulmonary TB and 9 cases of extra-pulmonary TB were reported to the Castellón Public Health Center. When a case of TB is reported, an epidemiological study of the case is carried out by the Epidemiology Division, and contacts of TB patients are invited to a medical examination that includes clinical, analytic and radiographic tests in order to control and prevent the disease.

The study had two phases. In phase I, contacts of TB patients were studied in the Epidemiology Division of the Public Health Center and the Preventive Medicine Service of Hospital La Plana (Vila-real). After an extensive interview, a TST with 2 TU (tuberculin units), equivalent to 5 IU (international units) of PPD (purified protein derivative RT-23) from the Serologic Institute of Copenhagen, was performed following the Mantoux technique. The test was read 72 hours later. A LTBI case was defined following a positive TST test result and negative clinical, analytic and radiographic examinations. The TST evaluation was considered positive in non BCG-vaccinated participants with an induration of ≥ 5 mm, or BCG-vaccinated participants with an induration of ≥ 15 mm or the presentation of vesicles [26]. After 7 days, a booster TST was conducted on participants older than 54 years or BCG-vaccinated with a negative first TST. Participants with a history of TB or with history of treatment for LTBI were considered positive LTBI. Participants with a negative TST were considered as controls in this phase. The extensive interviews with participants provided information on the nature of contacts (household members, family members, friends, work or study colleagues), and an average number of hours per day spent in close contact with the TB patient (high exposure: 6 or more hours/day, medium exposure: 1-5 hours/day, and low exposure: < 1 h/day). A self-report questionnaire gathered information on the participants' clinical history, demographic variables, weight and height, habits, use of VitD supplements, and other variables.

The serum level of 25 hydroxyvitamin D_3 _(25[OH]D) was measured by electrochemiluminescence immunoassay (ECLIA) on a COBAS^® ^410 ROCHE^® ^automate analyzer [27,28] in the Biochemical Laboratory of the Hospital General of Castellon. Serum 25(OH)D levels from 0-19 ng/ml range were considered as deficient, 20-29 ng/ml range as insufficient, and ≥ 30 ng/ml range as sufficient [29,30]. The analysis was performed on the day the TST was read, and laboratory staff were unaware of the TST results.

In phase II (case-control study), two months later, a new TST was performed on the people who tested LTBI negative in the first study. TST conversion in non BCG-vaccinated participants was considered as the change from negative to positive TST with an increase of ≥ 5 mm on the induration, and in BCG-vaccinated participants an increase of ≥ 10 mm on the induration from the initial TST, or the presence of vesicles, (26,31-32). New clinical, analytic and radiographic examinations were carried out to exclude active TB. Cases were the participants with positive TST conversion, and controls who presented negative TST results. Three participants received primary prophylaxis and were excluded from the case-control study, and all controls were contacts of pulmonary TB patients.

Considering the high percentage of BCG-vaccinated participants and the low specificity of TST, an alternative analysis was carried out. Participants with high exposure, sputum AFB-positive index cases and a TST with ≥ 5 mm of induration were defined as LTBI positive. Participants without this contact and a TST with ≥ 10 mm of induration were defined as LTBI positive [33] and BCG status was not considered. The same TST conversion criterion was used.

### Statistical methods

Qualitative variables were compared with Chi2 and Fisher tests; quantitative variables were compared using the Kruskal-Wallis test. Odds ratios (OR) with 95% confidence intervals (CI) were calculated by logistic regression. In the models, prevalent LTBI in phase I, and TST conversion in phase II were taken as dependent variables. Independent variables were demographic conditions and potential risk or protective factors. The independent variables associated with a p < 0.20 with dependent variables were included in the logistic regression models, to finally obtain those associated with a p < 0.05. The statistical programs used were STATA version 9 and LogXact [34,35].

The study was approved by the Hospital General of Castellón Ethics Committee, and signed informed consent was obtained from all the participants or their parents. Children younger than 6 years of age were not included in the study.

## Results

From October 2009 to November 2010, 62 TB cases were reported and 42 TB cases were included in the study, 28 cases of pulmonary TB with sputum acid-fast bacilli (AFB) positive smear and culture positive *Mycobacterium tuberculosis*, 8 cases of pulmonary TB with culture positive *M. tuberculosis *and sputum AFB-negative smear, 3 cases of TB pleurisy with culture negative *M. tuberculosis*, and 3 cases of lymph node TB (1 culture positive and 2 culture negative *M. tuberculosis*). The study population comprised 202 contacts of TB patients with a participation rate of 60.1% (202/336). The non-participants presented significant differences in the study variables, and were younger than participants, with a lower foreign-born proportion and less exposure and sputum AFB-positive index cases. Contacts of active TB patients were household members, family members, friends, and work or study colleagues. No active TB was found among the contacts.

Table 1 presents the description of the study population broken down into Spanish and foreign-born participants. The foreign-born participants came from Eastern Europe, the Maghreb, and Latin America. No differences were observed in relation to age, gender, or serum 25(OH)D levels. However, the foreign-born participants presented more sputum AFB-positive index cases, higher exposure, and a higher proportion of BCG vaccination than Spanish participants. Only 20.3% of the participants had a sufficient serum 25(OH)D level. All participants with serum 25(OH)D levels below 30 ng/ml were referred to a primary care physician with recommendations to take VitD supplements.

**Table 1:** Characteristics of the study population: Spanish and foreign-born participants

Variables	Spanish n = 119 (%)	Foreign-born n = 83 (%)
Age (Mean ± SD)	39.9 ± 14.3	32.9 ± 19.9
Male (%)	66 (55.5)	46 (55.4)
25 (OH)Vitamin D_3 _ng/ml (Mean ± SD)	23.8 ± 13.8	23.68 ± 12.9
Vitamin 25 (OH)D_3 _ng/ml (Mean ± SD) Oct-March period	19.5 ± 7.8	16.58 ± 7.41
Vitamin 25 (OH)D_3 _ng/ml (Mean ± SD) Apr-Sept period	25.7 ± 15.5	28.86 ± 13.6
25 (OH)VitaminD_3 _levels		
0-19 ng/ml	55 (46.2)	34(41.0)
20-29 ng/ml	38 (31.9)	34 (41.0)
≥ 30 ng/ml	26 (21.8)	15 (18.0)
Exposure to TB patient		
High	35 (29.4)	29(34.9)
Medium	51 (42.9)	44 (53.0)
Low	33 (27.7)	10 (12.1)
High exposure and sputum AFB-positive index cases	19 (15.9)	21 (25.3)
BCG vaccination	36 (30.3)	62 (74.7)
Tuberculosis types		
Pulmonary TB sputum positive AFB smear	81(68.1)	74 (89.9)
Pulmonary TB sputum positive culture *M. tuberculosis*	28 (23.5)	3 (3.6)
Pleurisy	7 (5.9)	2 (2.4)
Lymph node	3 (2.5)	4 (4.8)
Foreign-born population		
Eastern Europe		41 (49.4)
Maghreb		13 (15.7)
Latin America		29 (34.9)

SD = Standard deviation. BCG = bacilli Calmette-Guerin. AFB = Acid Fast Bacilli. TB = Tuberculosis

The results of the cross-sectional study are shown in Table 2. The prevalence of LTBI was 24.8% (50/202), and associated factors were male gender, exposure, and high exposure and sputum AFB-positive index cases. Preventive chemotherapy was indicated for 35 out of 50 participants with LTBI, after ruling out the disease. Three out of 14 participants with negative TST results, considering high exposure and sputum AFB-positive index cases, received primary prophylaxis with isoniazid for a two months period, after which one person presented TST conversion. These indications were independent of VitD status. Serum 25(OH)D levels were not associated with the prevalence of LTBI. However, sufficient 25(OH)D levels was a protector, although not significant (OR = 0.82 95% CI 0.36-1.87). The three participants, who received primary prophylaxis, had low 25(OH)D levels, 13.16, 22.4, and 22.72 ng/ml, respectively. In the multivariate analysis, LTBI prevalence was not associated with serum 25(OH)D levels.

**Table 2:** Prevalence of latent tuberculosis infection (LTBI) in the study population following the cross-sectional study

Variables	Cases	Controls	OR 95% CI	P value
		n = 50 (%)	n = 152 (%)	
Age (years) (Mean ± SD)	38.0 ± 16.9	34.8 ± 14.3	1.02 (0.99-1.04)	0.151
Male	35(70)	77 (50.7)	2.27 (1.15-4.50)	0.019
Foreign-born	22 (44)	61 (40.1)	1.17 (0.61-2.24)	0.630
BCG vaccinated	24 (48)	74 (48.7)	0.97 (0.51-1.84)	0.933
Use vitamin D supplements^1^	6 (13.6)	27 (18.0)	0.72 (0.28-1.87)	0.500
Ever smoking^2^	20 (46.5)	54 (38.3)	1.40 (0.70-2.79)	0.337
BMI^3^(Mean ± SD)	25.7 ± 4.7	25.6 ± 6.3	1.03 (0.97-1.11)	0.315
Sputum AFB-positive index cases	43 (86)	112 (73.7)	2.19 (0.91-5.27)	0.079
Exposure to TB patient				
High	28 (52)	36 (23.7)	7.58 (2.42-23.74)	0.001
Medium	18 (40)	77 (50.6)	2.28 (0.72-7.02)	0.160
Low	4 (8)	39 (25.7)	1.00	
Trend				0.000
High exposure and sputum AFB-positive index cases	23(46)	17 (11.2)	6.76 (3.19-14.33)	0.000
Study period Apr-Sept	32 (64)	98 (64.5)	0.98 (0.50-1.91)	0.952
^25 (OH)Vitamin D^3 ng/ml (Mean ± SD)	22.9 ± 11.0	24.0 ± 14.1		0.875^4^
^25 (OH)Vitamin D^3 ng/ml(Mean ± SD) Oct-March period	17.9 ± 7.0	18.2 ± 8.0		0.800^4^
^25 (OH)Vitamin D^3 ng/ml(Mean ± SD) Apr-Sept period	25.7 ± 11.9	27.2 ± 15.7		0.957^4^
^25(OH) Vitamin D^3 ≥ 30 ng/ml	9 (18)	32 (21.1)	0.82 (0.36-1.87)	0.642
^25 (OH)Vitamin D^3 levels				
0-19 ng/ml	22 (44)	67 (44.1)	1.00	
20-29 ng/ml	19 (38)	53 (34.9)	1.09 (0.54-2.22)	
30 ≥ ng/ml	9 (18)	32 (21.1)	0.86 (0.35-2.07)	
Trend				0.812

Comparison of positive LTBI cases and controls. Odds ratios (OR) and 95% confidence interval (CI).

(1) Information on 194 participants. (2) Information on184 participants. (3) Information on 174 participants. (4) Kruskal-Wallis test. SD = Standard deviation. BMI = Body mass index. AFB = Acid fast bacilli TB = tuberculosis.

In the second phase, the rate of participation was 69.6% (96/138) of the LTBI negative participants in phase I. Significant differences in the study variables were not found between participants and non-participants, except for the high proportion of foreign-born (59.5% versus 33.3% p = 0.004) non-participants. Twelve out of 96 participants (12.5%) presented TST conversion, and their characteristics are shown in Table 3. Seven patients were BCG-vaccinated, and all but one had sputum AFB-positive index case. All participants that presented TST conversion received preventive chemotherapy with isoniazid. These three participants with primary prophylaxis of TB were excluded of case-control study.

**Table 3:** Characteristic of participants with tuberculin skin test (TST) conversion

N°	Age	Gender	Foreign-born	Sputum AFB-positive index cases	Exposure to TB patient	BCG vaccine	Primary prophylaxis	TST 1ª	TST 2ª
1	6	Female	No	Yes	High	No	Yes	0	16
2	21	Female	Yes	Yes	High	No	No	0	12
3	22	Male	Yes	Yes	Medium	Yes	No	2	12
4	27	Male	No	Yes	High	No	No	2	9
5	34	Male	Yes	Yes	High	Yes	No	10	28
6	37	Male	No	Yes	High	No	No	0	30
7	38	Male	No	Yes	Medium	No	No	1	8
8	42	Female	Yes	Yes	High	Yes	No	0	20
9	46	Male	No	Yes	Medium	Yes	No	9	16^1^
10	55	Male	No	Yes	Medium	Yes	No	9	19
11	56	Female	Yes	No^2^	Medium	No	No	4	13
12	57	Female	No	Yes	Medium	No	No	0	7

(1) Presence of vesicles. (2) Contact with culture positive pulmonary TB patient.

AFB = Acid Fast Bacilli; BCG = Bacilli Calmette-Guerin; TST = Tuberculin Skin Test TB = Tuberculosis

In the case-control study, 11 cases with positive TST conversion and 82 controls with negative TST results participated. The results of the case-control study are shown in Table 4. High exposure to TB patient was associated with TST conversion (p = 0.014), and high exposure and sputum AFB-positive index cases presented strong association with TST conversion. The use of VitD supplements was not associated with TST conversion. All eleven TST conversion cases and 57 controls (69.5%) had low 25(OH)D levels, with means of 25(OH)D concentrations of 17.5 ± 5.6 ng/ml and 25.9 ± 13.7 ng/ml, respectively (p = 0.041). Seasonal variations in serum 25(OH)D levels were observed. A significant tendency (p = 0.034) was observed between TST conversion and 25(OH)D levels: 0-19 ng/ml, 20-29 ng/ml, and ≥ 30 ng/ml, with OR of 1.00, 0.60 (95% CI 0.12-2.66), and 0.13 (95% CI 0.00-0.91), respectively. When logistic regression models were performed, sufficient 25(OH)D level presented a significant inverse association with TST conversion (OR = 0.10 95% CI 0.00-0.76), adjusted for high exposure and sputum AFB-positive index cases with a significant tendency (p = 0.019) (see Table 5).

**Table 4:** Incidence of tuberculin skin tests (TST) conversion following the case-control study

Variables	Cases	Controls	OR 95% CI	P value
	N = 11(%)	N = 82 (%)		
Age (years) (Mean ± SD)	39.6 ± 13.1	34.7 ± 14.5	1.02 (0.98-1.07)	0.299
Male	8 (72.7)	41 (50.0)	2.07 (0.66-10.72)	0.168
Foreign-born	5 (45.5)	26 (31.7)	1.79 (0.50-6.42)	0.363
BCG vaccinated	5 (45.5)	40 (48.8)	0.88 (0.25-3.10)	0.886
Use vitamin D supplements	3 (27.3)	20 (24.7)^1^	1.14 (0.28-4.73)	0.853
Ever smoking	6 (54.5)	30 (38.5)^2^	1.96 (0.55-6.98)	0.299
BMI(Mean ± SD)^3^	25.4 ± 4.6	26.0 ± 6.4	0.98 (0.88-1.10)	0.749
Contact sputum AFB-positive index cases	10 (90.9)	65 (79.3)	2.62 (0.31-21.87)	0.375
Exposure to TB patient				
High	5 (45.5)	14 (17.1)	9.16 (1.14-∞)	0.035
Medium	6 (54.5)	47 (57.3)	3.51 (0.49-∞)	0.248
Low	0 (0.0)	21 (25.6)	1.00	
Trend				0.014
High exposure and sputum AFB positive index cases	5 (45.5)	6 (7.3)	10.56(2.48-44.95)	0.001
Study period Apr-Sept	6 (54.5%)	61 (74.4)	0.41 (0.11-1.50)	0.178
^25 (OH)Vitamin D^3 (ng/ml) (Mean ± SD)	17.5 ± 5.6	25.9 ± 13.7		0.041^4^
				
^25 (OH) Vitamin D^3 ng/ml (Mean ± SD) Oct-March period	15.3 ± 7.4	20.0 ± 9.3		0.242^4^
^25 (OH) Vitamin D^3 ng/ml (Mean ± SD) Apr-Sept period	19.4 ± 2.9	27.9 ± 14.4		0.144^4^
				
^25 (OH) Vitamin D^3 ≥ 30 ng/ml	0 (0)	25 (30.5)	0.15 (0.00-1.00)	0.050^5^
^25 (OH) Vitamin D^3 levels				
0-19 ng/ml	7 (63.6)	29 (35.4)	1.00	
20-29 ng/ml	4 (36.4)	28 (34.1)	0.60 (0.12-2.66)	0.660
≥ 30 ng/ml	0 (0.0)	25 (30.5)	0.13 (0.00-0.91)	0.038
Trend				0.034

Comparison of TST conversion cases and controls. Odds ratios (OR) and 95% confidence interval (CI).

(1) Information on 81 controls. (2) Information on 78 controls. (3) Information on 10 cases and 75 controls.(4) Kruskal-Wallis test. (5) Fisher test p = 0.033. SD = Standard Deviation. BCG = Bacilli Calmette-Guérin. BMI = Body Mass Index AFB = Acid Fast Bacilli. TB = Tuberculosis

**Table 5:** Risk and protective factors for tuberculin skin test conversion by logistic regression following the case-control study

Variables	OR	95% CI	P value
25 (OH)Vitamin D_3 _levels			
0-19 ng/ml	1.00		
20-29 ng/ml	0.49	(0.07-2.59)	0.563
≥ 30 ng/ml	0.10	(0.00-0.76)	0.024
Trend			0.019
High exposure TB and sputum AFB positive index cases	14.19	(2.15-120.64)	0.004

Odds ratios (OR) and 95% confidence interval (CI).

AFB = Acid Fast Bacilli. TB = Tuberculosis

Results of the alternative analysis revealed 10 participants out of 86 (11.6%) with TST conversion. The distribution of participants' serum 25(OH)D levels and TST conversion were 18.2%(6/33): < 20 ng/ml; 14.3% (4/28) 20-29 ng/ml; and 0.0% (0/25) ≥ 30 ng/ml, respectively. A significant tendency (p = 0.030) was found between 25(OH)D levels and TST conversion with OR of 1.00, OR = 0.58 (95% CI 0.08-3.24) and OR = 0.11 (95% CI 0.00-0.87), adjusted for high exposure and sputum AFB-positive index cases. The mean of 25(OH)D was 19.0 ± 2.9 ng/ml in cases versus 26.3 ± 14.0 ng/ml in controls (p = 0.094).

## Discussion

The results indicated that a high proportions of contacts of TB patients had low serum 25(OH)D levels, and suggested that sufficient 25(OH)D levels protect against TST conversion. In the cross-sectional study, no differences in 25(OH)D levels were found between negative and positive LTBI participants.

Some studies have found high prevalence of low serum VitD levels in various Spanish populations [36-38], but this is not general [39]. No Spanish studies of VitD in contacts of TB patients have been published. In the international research on VitD and TB, Gibney and co-authors [24] observed that higher VitD levels were associated with lower probability of LTBI and TB disease in immigrants from Sub-Saharan Africa in Melbourne, Australia. In Vietnam, Ho-Pham and co-authors [40], in a matched case-control study of TB patients and controls, found that low serum 25(OH)D levels(< 30 ng/ml) was a risk of tuberculosis in men, but not in women. In Greenland [41], a case-control study of TB patients and controls reported that 25(OH)D levels of < 75 nmol/l or > 140 nmol/l were associated with high risk for active TB. In Pakistan, a cohort study of household contacts of pulmonary TB patients found that VitD deficiency was a risk factor for active TB (Relative risk = 5.1 95% CI 1.2-21.3) [42], and this is consistent with our case-control study. Coinciding with phase I of our study, a cross-sectional study [25] of Mexican immigrants in Canada found no difference in 25(OH)D levels between positive LTBI cases and controls. In a cohort study of Greek dialysis patients, the use of VitD supplements was not associated with incidence of TB [43]. These studies vary in terms of design, geographic location and population, and some questions remain in relation to gender differences, the use of VitD supplements, and the effects of serum VitD levels.

The antimicrobial effects of VitD have motivated intense research [44]. In relation to *M. tuberculosis*, metabolites of VitD, calcitriol and the induced peptides, cathelicidin and defensina beta 4, have an important effect in eliminating the bacilli, including enhancement of macrophage phagocytosis, activation of monocytes, cytokines modulation, limitation of intracellular growth of *M. tuberculosis*, and suppression of enzymes involved in pulmonary cavitation [45-48]. In addition, the vitamin D receptor gene polymorphism plays a key role in susceptibility to TB [49].

The strengths of our study include the following: its design is prospective, it studied the contacts of recent diagnosed TB patients with actual exposure to *M. tuberculosis*, seasonal sunlight variations occurred during the study, multivariate analysis was applied to control potential confusion factors and risk of exposure, and a valid technique was used to determine 25(OH)D levels [50]. The study has some limitations, however. The size of the study in relation to TST conversion is small. Some potential confusion factors were studied but unknown factors could have been present. The measure of VitD status estimated 25-hydroxyvitamin D_3_, 90% of VitD circulating in blood, and not vitamin D_2_, and the technique has some discordant sensitivity at range limits when it is compared with radioimmunoassay [51]. There is no gold standard for the diagnosis of LTBI and the presence of live *M. tuberculosis *in LTBI or TST conversion patients is unknown [52]. The TST has low specificity with the possibility of false-positive results [53]. Two sources of false-positive results are *Non-tuberculous Mycobacteria *(NTMB) and BCG vaccination. In Spain, the frequency of NTMB is considered low, but the proportion of BCG-vaccinated people over the age of 40 is high, and interferon-γ release assays (IGRAS) tests were not used [54]. A booster TST was performed to deal with this issue. In addition, the incidence of TST conversion was in the range of results from several other studies of TB contacts in Spain, 7.8%-12.5% [55,56]. In the cross-sectional study, the participants presented significant differences from the non-participants, which is a limitation. However, the selection bias could be reduced, considering that participation might not be associated with VitD status. In the case-control study, only the foreign-born proportion of non-participants was significantly different. In addition, the participation rate in the case-control study was higher than the rates published in other studies in Spain, range 21.1%-63.3% [55]. Finally, no polymorphism study of VitD was carried out.

TB patients frequently have lower VitD levels than the general population [57] and research is still studying the effect of VitD on the course of the TB disease. In a recent clinical trial [48], the administration of VitD decreased the time of sputum culture conversion in pulmonary TB patients, but the decrease was only significant in patients with the group tt genotype of the TaqI vitamin D receptor polymorphism. In contrast, in another clinical trial [58] the administration of VitD did not improve clinical symptoms of TB patients. Our study indicates that it could be useful to determine 25(OH)D status in contacts of TB patients as a risk factor for TST conversion. If low 25(OH)D levels are found, recommendations to take Vit D supplements are pertinent in order to achieve sufficient 25(OH) D levels. However, only a clinical trial could determine the therapeutic effects of VitD in contacts of TB patients [59].

## Conclusion

The results of the study suggest that sufficient 25(OH)D levels protect against TST conversion and support the hypothesis that deficient VitD status is a TB risk factor.

## List of abbreviations

ABF: acid-fast bacilli; BCG: bacilli Calmette Guerin; IGRAS: interferon-γ release assays; IU: international units; LTBI: latent tuberculosis infection; PPD: purified protein derivative; SD: standard deviation; TB: tuberculosis; TST: tuberculin skin test; TU: tuberculin units; 25(OH)D: 25-hydroxyvitamin D_3_; VitD: vitamin D.

## Competing interests

The authors declare that they have no competing interests.

## Authors' contributions

AAP, and MARG designed the study; MARG, RHG, CHC, JBB, LSA, ESS, NMF and JIM carried out data collection and follow-up participants; LSA, ESS, and NMF performed the tuberculin skin test; JVJC, DGF, JIM, MJSP and JAFV performed determinations of 25 (OH) D; AAP, NMF, and ESS carried out the statistical analysis; AAP, AFV, RHG, VRB contributed to writing up the manuscript; JBB, JVJC, JAFV and RHG reviewed the manuscript. All authors read and approved the final manuscript.

## Pre-publication history

The pre-publication history for this paper can be accessed here:

http://www.biomedcentral.com/1471-2334/11/349/prepub
